# Predictive biomarkers for precision treatment in rheumatoid arthritis: are we there yet?

**DOI:** 10.3389/fphar.2026.1970975

**Published:** 2026-09-28

**Authors:** Jianyang Liu, Per-Johan Jakobsson

**Affiliations:** 1 Division of Rheumatology, Department of Medicine Solna, Center for Molecular Medicine, Karolinska Institutet, Karolinska University Hospital, Stockholm, Sweden; 2 Pharmacognosy, Department of Pharmaceutical Biosciences, Uppsala University, Uppsala, Sweden; 3 Medical Unit Rheumatology, Gastro-enterology and Dermatology, Karolinska University Hospital, Stockholm, Sweden

**Keywords:** biomarker, disease-modifying antirheumatic drugs, multi-omics, prediction, rheumatoid arthritis

## Abstract

Rheumatoid arthritis (RA) is a chronic autoimmune disease affecting approximately 1% of the global population and is associated with a substantial reduction in both life expectancy and quality of life. Although current treatments, primarily disease-modifying antirheumatic drugs, have been shown to suppress inflammation, clinical responses vary widely between individuals, reflecting the heterogeneity of the disease. Consequently, identifying reliable biomarkers to predict therapeutic response has become a key priority for improving clinical management. In this focused review, we summarise recent advances in the discovery of predictive biomarkers for RA and compare their reported specificity and sensitivity using published data. We also highlight key limitations in the existing literature that undermine reproducibility in this field. Coordinated efforts across the rheumatology community to address these challenges will be essential to translate biomarker research into personalised treatment strategies for RA.

## 1 Introduction

The treatment landscape for rheumatoid arthritis (RA) has advanced considerably, evolving from conventional disease-modifying antirheumatic drugs (DMARDs), such as methotrexate (MTX), to novel biological and targeted synthetic DMARDs, including tumour necrosis factor (TNF) inhibitors and Janus kinase (JAK) inhibitors. Despite these innovations, remission rates remain suboptimal; even the most effective JAK inhibitors achieve remission in only around 50% of patients. Furthermore, approximately 60% of patients fail to respond to individual biological DMARDs (bDMARDs), and up to 20% are refractory to all currently available DMARDs (Brown et al., 2024).

These limitations underscore two urgent needs in RA management: (i) the development of novel, disease-specific therapies to improve remission rates; and (ii) the identification of predictive biomarkers capable of predicting individual responses to specific DMARDs. While considerable efforts have been made to explore demographic and clinical predictors of treatment response, such as seropositivity, specific genetic variants, age, comorbidities, disease duration, Doppler severity, and smoking status, these factors have not demonstrated sufficient precision to guide treatment selection reliably (Lend et al., 2024; Crowson et al., 2024; Capelusnik and Aletaha, 2022; Gazel et al., 2025). As a result, no biomarkers are currently implemented in routine clinical practice for treatment stratification. In this focused narrative review, we summarise and discuss advances in biomarker research for treatment stratification in RA, focusing exclusively on biomarkers measurable before initiation of any DMARD therapy.

## 2 Literature search and selection

We searched PubMed using the following query: “rheumatoid arthritis” [Title/Abstract] AND (“predict” [Title/Abstract] OR “predicting” [Title/Abstract] OR “prediction” [Title/Abstract] OR “biomarker” [Title/Abstract] OR “associate” [Title/Abstract] OR “association” [Title/Abstract]) AND (“response” [Title/Abstract] OR “responder” [Title/Abstract] OR “responsiveness” [Title/Abstract] OR “effectiveness” [Title/Abstract] OR “remission” [Title/Abstract] OR “resistance” [Title/Abstract]). Abstracts were screened, and only publications directly relevant to the topic were reviewed further. The review includes English-language studies published from 1 January 2016 to 8 September 2026; earlier findings are summarised in previous comprehensive reviews (Plant et al., 2014; Schreiber et al., 2017; Priori et al., 2013).

This review focuses on predictive biomarkers for patient stratification; biomarkers relevant only at later stages, such as anti-drug antibodies, are therefore beyond its scope. Similarly, we exclude studies evaluating changes in biomarkers over time in relation to treatment response, as this information is unavailable at baseline and therefore has limited utility for initial treatment selection. Although inflammatory cytokines, such as TNF-α and interleukin-6 (IL-6), have been proposed as potential predictors of response to their corresponding inhibitors, these findings have generally lacked reproducibility and are also excluded. We further exclude studies relying solely on correlations (e.g., Pearson’s or Spearman’s) with reductions in C-reactive protein (CRP), erythrocyte sedimentation rate (ESR), or disease activity scores, and include only studies directly comparing biomarker levels between responders and non-responders. To assess clinical applicability, we exclude studies in which predictive accuracy cannot be evaluated because neither area under the curve (AUC) values nor sensitivity and specificity are reported or can be calculated from the available data. These criteria allow direct comparison of the accuracy of proposed biomarkers but may favour small studies reporting apparently strong classification performance, while omitting larger association studies reporting only odds ratios or hazard ratios. This selection bias represents a major limitation of this focused narrative review.

## 3 Genetic and epigenetic markers

Several studies have explored whether baseline genetic and epigenetic markers can be used to predict response to DMARDs. In one report, the authors developed multiple genomic based machine-learning models to predict response to TNF inhibitors (Guan et al., 2019). Using a combination of demographic, clinical and genetic variables measured at baseline, the models achieved accuracies exceeding 60% for predicting treatment response, significantly outperforming models based solely on baseline demographic and clinical parameters. Notably, the selected genetic parameters largely comprised single-nucleotide polymorphisms (SNPs) in genes involved in immunological processes. Moreover, their predictive contribution appeared to differ between DMARDs, implying that drug-specific biological pathways may influence treatment outcomes (Guan et al., 2019). Beyond SNPs, a large cohort study assessed the utility of genome-wide exome-derived potentially functional coding haplotypes for predicting MTX response (Lim et al., 2022). Responders and non-responders showed significant differences in haplotype profiles, and machine-learning models built on haplotype features predicted MTX response with AUCs ranging from 0.80 to 0.90 (Lim et al., 2022). While these findings are promising, this remains the only genome-wide exome analysis of this kind and therefore requires validation in independent cohorts before it can be considered sufficiently robust for translational use.

A complementary line of work has focused on higher-order chromatin architecture, commonly referred to as the epigenome. By comparing blood chromosome conformation signatures (CCS) between MTX responders and non-responders, investigators identified significant differences at loci including *IFNAR1*, *IL17A*, *CXCL13*, *IL21R* and *IL23* (Carini et al., 2018). Regression models built on these signals were reported to predict MTX response with an overall accuracy of around 0.75 in both discovery and validation cohorts (Carini et al., 2018). These findings suggest that structural genomic features may reflect immune-signalling processes, relevant to MTX efficacy.

DNA methylation has also been linked to treatment response, particularly to TNF inhibitors. For example, a higher frequency of hypermethylation in peripheral blood mononuclear cells (PBMCs) has been described among responders to etanercept (ETN) and adalimumab (ADA) (Tao et al., 2021). However, although methylation-based machine-learning models in that study reportedly achieved near-perfect predictive performance, such unusually high accuracies should be interpreted cautiously. They may reflect small sample sizes, overfitting, or cohort-specific technical effects rather than true generalisability (Tao et al., 2021). In addition, the limited overlap in differentially methylated sites between the ETN and ADA cohorts suggests that response-associated epigenetic signatures may be drug-specific rather than reflecting a shared TNF inhibition effect (Tao et al., 2021). This interpretation is supported by findings from a separate study of whole-blood methylation profile, which likewise suggested that response-associated signals may depend on the specific TNF inhibitor (Julià et al., 2022). By contrast, another cohort study comparing baseline whole-blood methylation profiles in ETN-treated patients reported greater hypermethylation among non-responders, with enrichment at exon 7 of *LRPAP1* (Plant et al., 2016). Nevertheless, the cohort was small (35 responders and 35 non-responders), and the authors did not develop multivariable predictive models combining data from the most significantly methylated sites (Plant et al., 2016). Altogether, the utility of DNA methylation profiles for treatment stratification remains uncertain, highlighting the need for rigorous validation in larger cohorts before these markers can be considered in routine clinical practice.

Another major concern is whether these genetic biomarkers perform consistently across populations of different ancestries. Most studies discussed above predominantly included participants of European ancestry (Guan et al., 2019; Carini et al., 2018; Plant et al., 2016), whereas one study was conducted in a Chinese population (Lim et al., 2022). Larger cohorts including participants from diverse ancestral backgrounds are needed to assess the generalisability of these genetic models for predicting response to DMARDs.

## 4 Transcriptomic markers

Tremendous effort has been dedicated to elucidating the relationship between the transcriptome and clinical response to DMARDs. Most investigations have focused on whole blood and PBMCs, largely because these samples are readily obtainable in routine practice. Among the various DMARDs, TNF inhibitors have been the most extensively investigated in this context.

In one study, significant differences were observed in genes associated with DNA and nucleotide binding within PBMCs when comparing responders and non-responders to TNF inhibitors (Tao et al., 2021). The researchers subsequently conducted cell-type–specific profiling in monocytes and CD4^+^ T cells. Notably, transcripts related to TNF signalling were upregulated in CD4^+^ T cells from responders, whereas gene expression patterns in monocytes showed relatively minor differences between the two groups. Leveraging these transcriptomic signatures, the authors developed several machine-learning models to predict response to TNF inhibitors, reporting near-perfect predictive performance within their datasets (Tao et al., 2021). Complementary to these finding, another investigation analysing whole-blood transcriptomes found that responders exhibited a more attenuated adaptive immune signature at baseline compared to non-responders (Yap et al., 2025). A prediction model consisting of the 30 most differentially expressed genes achieved an AUC of 0.86. Interestingly, network-based analysis identified a key node, *MZB1*, which was strongly associated with non-responsiveness to TNF inhibitors, potentially implicating a biologically relevant pathway in treatment failure (Yap et al., 2025). Another similar study on whole-blood transcriptome proposed a seven-gene panel, including *MRPL24*, *COMTD1*, *DNTTIP1*, *GLS2*, *GTPBP2*, *IL18R1*, and *KCNK17*, to distinguish responders from non-responders (Santiago-Lamelas et al., 2025). This model was validated across three independent cohorts, predominantly consisting of patients with an inadequate response to MTX, and demonstrated remarkably high predictive performance, with AUC values exceeding 0.9, whereas serological parameters showed no predictive value (Santiago-Lamelas et al., 2025). Lastly, a focused study on infliximab (IFX) identified the *RAC1*–*PAK1* signalling axis as commonly hyperactivated in responders (Gerassy-Vainberg et al., 2023). A pathway score calculated based on expression levels of six core genes, including *RAC1*, *PAK1*, *LYN*, *ICAM1*, *IL1B*, and *FCGR3A*, was able to predict non-responsiveness to IFX with an AUC of 0.7 (Gerassy-Vainberg et al., 2023). Despite the promising findings reported, it is notable that, although these studies employed broadly similar approaches to identify predictive gene expression profiles associated with TNF inhibitor therapy, none of the differentially expressed genes identified in one study have been successfully replicated in others. This lack of reproducibility highlights a major limitation in omics-based biomarker research in RA (Triaille et al., 2025).

Rather than relying on baseline profiles, one group assessed transcriptomic changes following exposure to TNF inhibitors in PBMC culture. They reported that, among responders, but not non-responders, ETN and ADA significantly increased expression of *PTPN22* and its related pathway genes, including *PFKFB3*, *ATM*, and *IL-17F* (Chang et al., 2019). Using a fold-change cut-off of 1.4, the increase in *PTPN22* expression by TNF inhibitors *in vitro* discriminated responders from non-responders with accuracy approaching 100% in the discovery cohort (Chang et al., 2019). Because this initial analysis included only 14 patients, the authors then validated the finding in a larger cohort comprising 28 responders and 11 non-responders (Ho et al., 2021). Although the validation cohort again showed a greater ADA/ETN-induced increase in *PTPN22* among responders, discrimination was more modest, with an AUC of around 0.75 (Ho et al., 2021).

Beyond TNF inhibitors, conventional synthetic DMARDs (csDMARDs) have also been widely studied. In one study investigating whether whole-blood transcriptomics could predict response to MTX, genes involved in type I interferon (IFN) responses and the type I IFN signalling pathway were significantly upregulated in non-responders (Plant et al., 2019). Logistic regression models based on the whole-blood transcriptome and on IFN-pathway transcripts achieved AUCs of approximately 0.7 (Plant et al., 2019). The authors also constructed a prediction model incorporating demographic and clinical variables, including sex, age, HAQ, smoking, ACPA positivity, swollen joint count, tender joint count, CRP, and VAS. This model was substantially less accurate than the transcriptomic model (Plant et al., 2019). The association between IFN signalling and poor response to csDMARDs has been verified in another study, where baseline IFN gene signature (IGS) was negatively associated with the probability of achieving a good EULAR response, prompting the authors to propose IGS as a predictive biomarker for csDMARD response (Cooles et al., 2022). However, when patients achieving disease activity score-28 (DAS28) remission were compared directly with those who remained with active disease after treatment, baseline IGS did not differ significantly. In addition, circulating interferons (IFN-α, IFN-β, and IFN-γ) were not associated with clinical outcomes (Cooles et al., 2022). These findings indicate that the predictive value of IFN-pathway transcripts remains unclear and warrants further validation in larger cohorts.

Other studies have attempted to develop a universal transcriptomic marker to predict response to DMARDs, regardless of mechanism of action. For example, Yamada and colleagues analysed transcriptomic profiles across 18 peripheral blood immune cell subsets and reported that gene expression signatures in dendritic cell (DC) precursors were significantly lower in DMARD responders than in treatment-resistant patients (Yamada et al., 2023). Despite the small sample size, the authors suggested that this difference was independent of DMARD class. This expression signature, termed pDC_M18, was negatively correlated with IFN-α and IFN-γ expression and showed no association with demographic or clinical parameters. Direct comparison with ACPA and disease duration showed that the pDC_M18 signature provided better discrimination between responders and non-responders (Yamada et al., 2023). In addition, DMARD treatment did not appear to alter pDC_M18 scores (Yamada et al., 2023). Another study has focused on the relationship between mitochondrial gene expression and treatment outcomes in RA. Using data extracted from four previously published cohorts, the authors stratified patients into three clusters based on mitochondrial transcriptomic profiles. A cluster characterised by lower activation of inflammatory pathways demonstrated a more favourable response to DMARD therapy (Wang et al., 2023). However, this study has two major limitations: definitions of “response” varied substantially across cohorts, and the analysis did not adjust for baseline inflammation or disease activity.

In addition to sequencing whole blood and PBMCs, several studies have investigated whether the synovial transcriptome is associated with response to DMARDs. Much of the work in this area has been led by Costantino Pitzalis’s group at QMUL, using data generated from three cohorts: R4RA, PEAC, and STRAP.

In their first report, the authors examined differences in synovial pathotypes and synovial tissue transcriptomes between responders and non-responders to csDMARDs in the PEAC cohort (Humby et al., 2019). They observed that remission rates were broadly similar across pathotypes. Nevertheless, integrating pathotype information with synovial transcriptomic profiles improved prediction of radiographic outcomes, defined by one-year changes in the van der Heijde modified Sharp score (SHSS) (Humby et al., 2019). In contrast, a subsequent analysis from the same group using the same cohort reported that patients with the lympho-myeloid pathotype were most likely to require escalation to bDMARDs (55%), compared with those with pauci-immune (21%) or diffuse-myeloid (21%) pathotypes (Lliso-Ribera et al., 2019). At the transcriptomic level, they identified 119 genes that were differentially expressed between csDMARD responders and non-responders. Moreover, a logistic regression model incorporating synovial pathotypes, clinical variables, and 13 of the most strongly differential genes predicted the need for additional bDMARDs with an AUC approaching 0.8 (Lliso-Ribera et al., 2019). By comparison, models incorporating only clinical variables and synovial pathotypes showed weaker predictive performance, with AUCs of around 0.75 (Lliso-Ribera et al., 2019). Importantly, the similarities and differences between these two studies were not discussed in detail, and the extent of overlap in the study populations between the two publications remains unclear (Humby et al., 2019; Lliso-Ribera et al., 2019).

The group has also explored relationships between the synovial transcriptome and response to several bDMARDs, including rituximab (RTX), ETN, and tocilizumab (TCZ). Initially, they reported that the diffuse-myeloid pathotype was associated with a poorer response to RTX (Rivellese et al., 2022). Bulk RNA sequencing of synovial samples further suggested that *HOX* genes and other leucocyte-related genes were linked to better responses to both RTX and TCZ, whereas fibroblast-associated genes correlated with poorer responses to both agents (Rivellese et al., 2022). To improve predictive performance, the authors subsequently developed machine-learning models integrating transcriptomic features, reporting AUC values of approximately 0.7 (Rivellese et al., 2022). The authors also evaluated models incorporating age, sex, CDAI, DAS28, CRP, ESR, TJC/SJC, and histology, which showed no predictive value. Consequently, no clinical features were retained in the final predictive model (Rivellese et al., 2022). In a more recent study, they extended a similar approach to include the TNF inhibitor ETN (Lewis et al., 2025). Notably, they found that differential expression profiles between responders and non-responders comprised both shared and treatment-specific signatures: genes commonly upregulated in responders were enriched for B-cell activation pathways, while non-response was associated with genes involved in fatty acid metabolism (Lewis et al., 2025). However, although B-cell activation signatures were linked to better response, B-cell abundance itself was not predictive of treatment outcomes (Lewis et al., 2025; Rivellese et al., 2023). In analyses of cellular composition by single-cell RNA sequencing (scRNA-seq), the authors also reinforced their earlier observation that sublining fibroblasts were strongly associated with treatment response to bDMARDs (Rivellese et al., 2022; Lewis et al., 2025). Using combinations of clinical and gene-expression variables, they built several predictive models for response to different bDMARDs, with reported AUC values around 0.75 (Lewis et al., 2025). The full model was compared with models based solely on clinical variables, which achieved AUCs of around 0.6 (Lewis et al., 2025). To facilitate clinical translation, they then adapted RNA-seq–based models to a NanoString platform, reporting overall accuracies of up to 80%. In addition, they validated these models in an independent cohort, achieving overall accuracies of 0.6–0.8 for predicting response to RTX and TCZ (Lewis et al., 2025). Despite these promising results, the authors did not fully address the similarities and differences between the two studies. Moreover, a manual comparison of the differentially expressed genes reported for responders versus non-responders suggests limited overlap between these publications (Rivellese et al., 2022; Lewis et al., 2025). Taken together, these issues raise concerns about the reproducibility of the findings and the robustness of the proposed predictive models.

Nevertheless, the association between specific fibroblast populations and poor response to RTX and TCZ appears to be supported by independent evidence, as another group reported similar findings using scRNA-seq of RA synovium (Zhang et al., 2023). In this study, 77 distinct cell states were identified, comprising 24 T-cell subsets, 9 B-cell subsets, 14 NK-cell subsets, 15 myeloid subsets, 10 stromal subsets, and 5 endothelial subsets. By integrating these data, the authors defined six “cell-type abundance phenotypes” (CTAPs), including CTAP-EFM, CTAP-F, CTAP-TF, CTAP-TB, CTAP-TM, and CTAP-M, which were associated with histological features and cytokine profiles but not with baseline clinical parameters. Of particular interest, CTAP-F, which is characterised by fibroblast-enriched patterns, including *PRG4* ^+^ *CLIC5* ^+^ lining cells, *PRG4* ^+^ lining cells, and CD34^+^ sublining cells, was associated with poorer response to biological DMARDs (Zhang et al., 2023). However, given the relatively low proportion of patients classified as CTAP-F (approximately 20%), its sensitivity as a stratification marker is likely to be limited. Moreover, these findings were derived from the same R4RA cohort used in the studies from Pitzalis’s group (Rivellese et al., 2022; Lewis et al., 2025) and therefore require validation in independent cohorts.

## 5 Protein and proteomic markers

Several studies have explored whether treatment response to DMARDs can be predicted using proteins measured in blood samples. The most frequently investigated candidates are IFNs and proteins involved in IFN signalling, reflecting the strong associations reported in transcriptomic analyses (Plant et al., 2019; Cooles et al., 2022; Cooles and Isaacs, 2022). In one study assessing links between IFN levels and treatment outcomes, patients with higher IFN-γ and lower IFN-α2 levels were more likely to achieve remission (Fukui et al., 2025). Nevertheless, the predictive performance was inadequate for routine clinical use. By contrast, another study suggested that the IFN-β/α ratio, rather than total type I IFN levels, may predict non-responsiveness to TNF inhibitors (Wampler Muskardin et al., 2016). Using a cut-off of 1.3, the IFN-β/α ratio achieved a sensitivity of approximately 0.65 and a specificity of around 0.9. However, both sensitivity and specificity declined markedly in a larger validation cohort, although a significant difference in the ratio between responders and non-responders remained (Wampler Muskardin et al., 2016).

Beyond IFNs, other inflammatory markers have been evaluated for predictive utility, including calprotectin. One study first reported that neutrophil activation markers were significantly higher in responders than in non-responders to the JAK inhibitor baricitinib, based on whole-blood RNA sequencing (Kuley et al., 2024). The investigators subsequently measured plasma calprotectin (the S100A8/A9 complex) and NET-DNA complexes, showing that both were increased in responders (Kuley et al., 2024). However, S100A8/A9 has also been proposed as a predictor of response to other DMARDs, including TNF inhibitors, and these findings have not been consistently replicated in subsequent studies (Smith et al., 2017; Choi et al., 2015). In a separate line of work, the potential of the multi-biomarker disease activity (MBDA) score to predict response was examined in patients who failed to achieve clinical remission on MTX monotherapy and were subjected to either triple therapy (MTX + sulfasalazine (SSZ) + hydroxychloroquine (HCQ)) or TNF inhibition (MTX + IFX) (Hambardzumyan et al., 2017). The MBDA score incorporates acute-phase reactants as well as more RA-specific serum markers, including inflammatory cytokines, cell-adhesion molecules, and matrix metalloproteinases. Patients with low MBDA scores had a substantially higher probability of achieving remission on triple therapy (79% vs. 36%), and this association was reported to be independent of seropositivity. Compared with stratification based on CRP, ESR, DAS28, or serostatus, MBDA scores better predicted differential treatment response to guide the selection of triple therapy (Hambardzumyan et al., 2017). In contrast, MBDA scores did not predict response to TNF inhibition in the same study (Hambardzumyan et al., 2017). Similarly, in a study of patients treated with ADA or abatacept (ABA), MBDA scores were again unable to predict clinical response (Fleischmann et al., 2016).

Proteomic approaches have also been used to identify candidate predictive biomarkers in blood. One serum proteomics study reported higher levels of epididymis secretory protein, testicular tissue protein, and the fibrinogen beta chain in responders to triple therapy (MTX + leflunomide (LEF) + IFX) compared with non-responders, whereas conventional inflammatory markers, such as CRP, did not differ significantly between the groups (Chen et al., 2019). However, the investigators did not attempt to combine these proteins into a predictive model, and the small cohort size was another major limitation of the study (Chen et al., 2019). Another study found that responders to MTX + LEF had higher plasma levels of *LGALS3BP*, *MYH9*, and *CBR1*, alongside lower levels of *COL1A1* and *ECI2*. Meanwhile, responders to MTX + HCQ showed higher levels of *RPL27A* and lower levels of *GGT1* (He et al., 2025). Notably, the limited overlap in differentially abundant proteins between these two commonly used csDMARD strategies suggests that the circulating proteome alone may be insufficient to predict response to MTX itself (He et al., 2025). Using LASSO-based models, the authors reported AUC values of around 0.9 for predicting response to MTX + LEF and MTX + HCQ (He et al., 2025). Clinical parameters such as VAS, SJC, TJC and CRP were also tested in combination to the proteomic features, however they did not seem to improve the performance with modestly increased AUC (He et al., 2025). However, these findings should be interpreted with caution, because the cohort was relatively small, ELISA-based validation did not fully replicate the proteomic results, and the definition of “response” was not clearly specified (He et al., 2025).

## 6 Metabolomic markers

Compared with genomic, transcriptomic and proteomic research, far less work has examined whether metabolomic features can predict treatment response. Nonetheless, a recent study reported that responders and non-responders to csDMARDs had significantly different baseline metabolomic profiles (Zhu et al., 2025). Regression models built from the leading candidate metabolites effectively discriminated responders from non-responders to MTX + LEF and MTX + HCQ, achieving AUCs of 0.83 and 0.92, respectively. By contrast, models based solely on clinical features showed no predictive value (Zhu et al., 2025). Notably, the metabolites that differed between responders and non-responders in these two treatment arms did not overlap, which suggests that the models may be capturing response to LEF and HCQ rather than to MTX itself (Zhu et al., 2025).

Rather than screening the entire metabolome, another study focused on a specific lipid class—oxylipins. In that analysis, baseline concentrations of several prostanoids and pro-resolving lipid mediators, including resolvins and maresins, were higher in csDMARD non-responders (Gomez et al., 2020). Using a predictive model comprising the leading oxylipin candidates, the authors reported an accuracy above 0.8 for predicting a good response to csDMARDs. By contrast, a random forest model based solely on clinical parameters showed no predictive value, with an AUC of around 0.5 (Gomez et al., 2020). Furthermore, when stratifying by pathotype, the model appeared particularly informative in the fibroid subgroup, with accuracies of 87% and 89% for predicting non-response and response, respectively (Gomez et al., 2020).

However, as in other omics studies, the reproducibility of metabolite- and oxylipin-based predictors remains limited. For instance, two metabolomic studies comparing MTX responders and non-responders identified 9 and 19 significantly different metabolites, respectively, yet there was no overlap between the proposed biomarkers (Fatima et al., 2025; Medcalf et al., 2022). In addition, the oxylipin study has been criticised on methodological grounds, with evidence suggesting that the measured signals largely reflected systemic noise and that the proposed pro-resolving lipid mediators were not present at the reported concentrations (O'Donnell et al., 2023). More broadly, the analysis of circulating oxylipins, including prostanoids, is well recognised as challenging and difficult to reproduce because of their rapid metabolism. An independent study that sought to validate the predictive value of urinary prostanoids found comparable levels in responders and non-responders across several DMARDs, casting further doubt on the reproducibility of the original findings (Liu et al., 2024).

## 7 Cell markers

In addition to multi-omics-derived biomarkers, several groups have investigated potential blood-based cellular markers that could predict treatment response. Among circulating immune cells, T cells have emerged as the most consistently implicated candidates. One study reported that non-responders had higher proportions of T cells producing TNF, IFN-γ and IL-2 than responders at baseline (Onofrio et al., 2018). By contrast, a separate study suggested that responders to TNF inhibitors had a greater abundance of circulating GM-CSF–expressing T cells (Bystrom et al., 2017). Nevertheless, the discriminatory power of these T-cell readouts appears insufficient for clinical implementation. Further work has examined whether peripheral T cells predict response to MTX. In one cohort, non-responders had a lower proportion of CD4^+^ T cells and a correspondingly higher proportion of CD8^+^ T cells. Consequently, the baseline CD4/CD8 ratio was approximately 50% higher in responders (Slauenwhite et al., 2020). More detailed phenotyping indicated that non-responders also exhibited increased frequencies of ICOS+ CD4^+^ regulatory T cells and IL-13^+^ CD4^+^ effector memory T cells. Notably, both the CD4/CD8 ratio and IL-13^+^ CD4^+^ effector memory T cells at baseline predicted MTX non-responsiveness with an AUC of around 0.75 (Slauenwhite et al., 2020). Evidence linking T cells to DMARD response has also been reported in a study profiling immune subsets in both peripheral blood and synovial tissue, including B cells, T cells, monocytes and DCs. In the analysis, synovial PD-1^+^ Tph/Tfh cells were associated with a higher likelihood of achieving DAS28 remission (Rivellese et al., 2025). However, the statistical support was borderline (with a P value close to 0.05) and the predictive effect modest. Moreover, the authors applied remission criteria that differed from those used in their earlier publications on the same cohort, which further weakens confidence in the robustness of the finding (Humby et al., 2019; Lliso-Ribera et al., 2019). A more recent study, however, reported that higher levels of Tph/Tfh cells were associated with a lower response rate to ETN (Xu et al., 2026).

Beyond T cells, pre-DCs have also been proposed as predictors of non-responsiveness to ABA, whereas several other immune populations, including multiple B and T cell subsets, did not differ between responders and non-responders. Despite improved sensitivity and specificity, these results were derived from a relatively small cohort and therefore require validation in independent, adequately powered studies (Yamada et al., 2023).

## 8 Precision medicine in RA-the unmet need

In summary, despite considerable efforts to identify biomarkers that predict response to DMARD therapy, none has been consistently validated across independent cohorts, and there remains no reliable predictive biomarker ready for routine clinical use. Much of the current literature is limited by small sample sizes (with statistical power rarely addressed), the absence of validation cohorts, suboptimal statistical approaches (such as a failure to adjust for baseline disease activity) and a lack of disclosure regarding cohort reuse. Moreover, reproducibility in this field is strikingly poor. For instance, transcriptomic studies have proposed entirely different candidate genes for predicting response to TNF inhibitors (Tao et al., 2021; Yap et al., 2025; Santiago-Lamelas et al., 2025).

This lack of reproducibility is likely multifactorial and reflects, among other issues, the methodological limitations outlined above. Notably, one of the most influential contributors is the inconsistent definition of “response” across studies, as summarised in Table 1. In some cases, even the same research group analysing the same cohort has applied different response definitions (Rivellese et al., 2022; Lewis et al., 2025). In addition, some studies did not specify how response was defined at all (He et al., 2025). Furthermore, more than half of the studies reviewed in this review combined patients treated with multiple DMARDs and attempted to identify a “universal” predictive biomarker. This approach is problematic because DMARDs act through distinct mechanisms, making a single biomarker that predicts response across all therapies unlikely on biological grounds. Consequently, it is unsurprising that studies on mixed or unspecified treatments tend to show poor reproducibility.

**TABLE 1:** Summary of suggested predictive biomarkers.

Suggested biomarkers	Study cohort (if applicable)	DMARDs^a^	Study size (N)^b^	Identification of responders/non-responders (N)^b^	Type of validation^c^	Definition of response	Sen^d^ ^,^ ^e^	Spe^d^ ^,^ ^e^	AUC (with 95% CI if available)^e^
Genomics and epigenetics									
Gaussian process regression models consisting of demographic, clinical and genetic parameters (Guan et al., 2019)	DREAM; CERTAIN	ADA; ETN; IFX	1892 (E); 680 (V)	Non-responders (436 (E); 238 (V))	External	ΔDAS28 at 12 months (unknown cutoff)	​	​	0.527 (V, ADA); 0.638 (V, ETN); 0.599 (V, IFX)
6 different MLMs based on 95 coding haplotypes and 5 nongenetic factors from whole blood exome sequencing (Lim et al., 2022)	​	MTX; MTX + SSZ; MTX + HCQ	279 (E); 70 (V)	Responders (161 (E); 32 (V))	Internal	DAS28 < 2.6 at 2 years	0.74–0.83 (O, E); 0.66–0.81 (O, V)	0.77–0.87 (O, E); 0.68–0.87 (O, V)	0.82–0.91 (E); 0.78–0.83 (V)
Logistic regression models based on chromosome conformation signature profiles in blood (Carini et al., 2018)	SERA	MTX	67 (E); 19 (V)	Responders (34 (E); 9 (V))	Internal	EULAR response at 6 months	0.67 (C, E); 0.67 (C, V)	0.87 (C, E); 0.85 (C, V)	0.9 (E); 0.91 (V)
Random forest models based on methylation profiles in PBMCs (Tao et al., 2021)	BiOCURA	ADA	38 (E); 4 (V)	Non-responders (18 (E); 3 (V))	Internal	EULAR response at 6 months	1.0 (C, E); 1.0 (C, V)	1.0 (C, E); 0.75 (C, V)	​
		ETN	42 (E); 5 (V)	Non-responders (23 (E); 2 (V))			1.0 (C, E); 1.0 (C, V)	1.0 (C, E); 0.66 (C, V)	​
DNA methylation levels at LRPAP1 gene in whole blood (Plant et al., 2016)	BRAGGSS	ETN	72 (E)	Responders (36 (E))	No validation	EULAR response at 3 months	0.66 (E, E)	0.95 (E, E)	​
Transcriptomics (Bulk/Single-cell sequencing)									
MLMs based on PBMC and CD4^+^ T cell transcriptome (Tao et al., 2021)	BiOCURA	ADA	38 (E); 4 (V)	Non-responders (18 (E); 3 (V))	Internal	EULAR response at 6 months	1.0 (C, E); 1.0 (C, V)	1.0 (C, E); 0.75 (C, V)	​
		ETN	42 (E); 5 (V)	Non-responders (23 (E); 2 (V))			1.0 (C, E); 1.0 (C, V)	0.93 (C, E); 1.0 (C, V)	​
Random forest models consisting of gene expression data from whole blood cells (Yap et al., 2025)	BRAGGSS	ADA; MTX + ADA	97 (E)	Responders (70 (E))	No validation	EULAR response at 6 months	​	​	0.86 (E)
Logistic regression models consisting of expression levels of 7 genes in PBMC and whole blood cells (Santiago-Lamelas et al., 2025)	GSE33377; GSE138746; COMBINE	ADA; CZP; ETN; GOL; IFX	122 (E); 279 (V)	Responders (57 (E); 169 (V))	Extremal	EULAR response at 3–6 months	0.87 (O, E)	0.95 (O, E)	0.95 (E); 0.85–0.94 (V)
RAC1-PAK1 pathway score consisting of expression data on 6 genes from leukocyte transcriptome (Gerassy-Vainberg et al., 2023)	GSE20690; GSE33377; GSE42296	IFX	129 (E)	Non-responders (55 (E))	RA as a different disease validation	EULAR response at 14 weeks	​	​	0.72 (E)
Fold increase in PTPN22 expression by TNFi in anti-CD3 stimulated PBMCs *in vitro* (cutoff 1.4 fold change)	CPIRA; PROSET-HD (Chang et al., 2019)	ADA; ETN	14 (E)	Responders (9 (E))	Exploration (Chang et al., 2019) and validation (Ho et al., 2021) in separate papers	ΔCDAI at 12 months (unknown cutoff)	1.0 (C, E, ADA); 1.0 (C, E, ETN)	1.0 (C, E, ADA); 1.0 (C, E, ETN)	​
	TARGET (Ho et al., 2021)		39 (V)	Responders (28 (V))		ΔCDAI≥12/6 at 12 months	0.86 (C, V, ADA); 1.0 (C, V, ETN)	0.55 (C, V, ADA); 0.41 (C, V, ETN)	0.75 [0.57–0.95] (V)
L2-regularized logistic regression model based on whole blood transcriptome (Plant et al., 2019)	RAMS	MTX	85 (E)	Non-responders (43 (E))	No validation	EULAR response at 6 months	​	​	0.66 (E)
Generalised linear model consisting of 207 genes in peripheral pre-DCs (Yamada et al., 2023)	​	ABA; MTX; TCZ; TFB	55 (E); 19 (V)	Non-responders (18 (E); 10 (V))	External	CDAI 50% response at 6 months	​	​	0.87 (E)
A cluster of patients defined by expression levels of 88 mitochondrial genes (Subtype C) (Wang et al., 2023)	GSE110169; GSE93272; GSE58795; GSE15258; GSE37107; GSE68215	ABA; IFX; MTX; RTX; unspecified TNF inhibitors	155 (E)	Responders (101 (E))	No validation	Unspecified	0.38 (C, E, RTX); 0.41 (C, E, ABA/MTX); 0.24 (C, E, anti-TNF)	1.0 (C, E, RTX); 0.95 (C, E, ABA/MTX); 0.82 (C, E, anti-TNF)	​
Logistic regression model including synovial pathotypes, clinical parameters and 3 gene expression levels from synovial tissue (Humby et al., 2019)	PEAC	Unspecified csDMARDs	75 (E)	Non-responders (14 (E))	No validation	ΔSHSS ≥1 at 1 year	​	​	0.88 (E)
L1-regularized logistic regression model including synovial pathotypes, clinical parameters and 13 gene expression levels from synovial tissue (Lliso-Ribera et al., 2019)			153 (E)	Non-responders (39 (E))		DAS28 < 3.2 at 6 months	​	​	0.81 (E)
Elastic-net regression models using transcriptomic data of leukocyte and fibroblast-specific genes (Rivellese et al., 2022)	R4RA	RTX	89 (E)	Responders (45 (E))	No validation	CDAI 50% response at 16 weeks	​	​	0.74 (E)
		TCZ	97 (E)	Responders (56 (E))			​	​	0.68 (E)
MLMs (Elastic net regression for ETN; Gradient boosted machine for TCZ; Extreme gradient boosting, linear booster for RTX) consisting of clinical and transcriptomic data from synovial biopsies (Lewis et al., 2025)	STRAP; R4RA	ETN	67 (E)	Responders (34 (E))	No validation	DAS28-ESR <3.2 at 16 weeks (STRAP); CDAI 50% response at 16 weeks (R4RA)	0.73 (C, E)	0.70 (C, E)	0.76 (E)
		TCZ	69 (E); 65 (V)	Responders (48 (E); 30 (V))	External		0.83 (C, E); 0.4 (C, V)	0.57 (C, E); 0.94 (C, V)	0.75 (E); 0.71 (V)
		RTX	72 (E); 68 (V)	Responders (21 (E); 16 (V))	External		0.67 (C, E); 0.56 (C, V)	0.77 (C, E); 0.83 (C, V)	0.75 (E); 0.79 (V)
Cell-type abundance phenotype-Fibroblast (CTAP-F) (Zhang et al., 2023)	R4RA	RTX	68 (E)	Non-responders (39 (E))	No validation	CDAI 50% response at 16 weeks	0.18 (C, E)	0.81 (C, E)	​
		TCZ	65 (E)	Non-responders (28 (E))			0.25 (C, E)	0.78 (C, E)	
Proteomics/Proteins									
IFN-γ high (>28.4 pg/mL)/IFN-α2 low (<20.0 pg/mL) group (Fukui et al., 2025)	KUDOS	Unspecified DMARDs	200 (E)	Responders (77 (E))	No validation	CDAI remission at 12 months	0.30 (C, E)	0.88 (C, E)	​
IFN-β/α activity ratio (>1.3) (Wampler Muskardin et al., 2016)	ABCoN; TETRAD	ADA; CZP; ETN; GOL; IFX	32 (E); 92 (V)	Non-responders (13 (E); 44 (V))	External	EULAR response at 12–14 weeks	0.67 (O, E); 0.45 (O, V)	0.92 (O, E); 0.77 (O, V)	​
Neutrophil activation score based on calprotectin (S100A8/A9) and NETs-DNA complex levels (Kuley et al., 2024)	RA-BEACON	BARI	220 (E)	Responders (124 (E))	No validation	ACR20 response at 24 weeks	0.98 (O, E)	0.11 (O, E)	0.55 (E)
Multi-biomarker disease activity score (cutoff = 38) consisting of 12 serum biomarkers (Hambardzumyan et al., 2017)	SWEFOT	MTX + SSZ + HCQ	75 (E)	Responders (35 (E))	No validation	DAS28 < 3.2 at 12 months	0.43 (C, E)	0.90 (C, E)	​
Serum proteome (FGB, HEL-S-282, HTL 70) (Chen et al., 2019)	​	MTX + LEF + IFX	20 (E)	Responders (15 (E))	No validation	EULAR response at 14 weeks	​	​	FGB: 0.720 (E); HEL: 0.853 (E); HTL: 0.787 (E)
L1-regularized logistic regression model consisting of 2 proteins (He et al., 2025)	Likely overlap with (Zhu et al., 2025)	MTX + HCQ	37 (E); 46 (V)	Responders (13 (E); 30(V))	External validation	EULAR response at 3–6 months	0.63 (V)	1.0 (V)	0.82 (E); 0.86 (V)
L1-regularized logistic regression model consisting of 5 proteins (He et al., 2025)		MTX + LEF	53 (E); 19 (V)	Responders (19 (E); 11 (V))			0.82 (V)	1.0 (V)	0.88 (E); 0.90 (V)
Metabolomics									
L2-regularized logistic regression model consisting of 9 plasma metabolites and 3 clinical parameters (Zhu et al., 2025)	Likely overlap with (He et al., 2025)	MTX + LEF	73 (E)	Responders (30 (E))	No validation	EULAR response at 3–6 months	​	​	0.83 [0.61–0.96] (E)
L2-regularized logistic regression model consisting of 6 plasma metabolites and 2 clinical parameters (Zhu et al., 2025)		MTX + HCQ	47 (E)	Responders (20 (E))			​	​	0.92 [0.73–0.98]
Random forest model consisting of 4 specialised pro-resolving lipid mediators in plasma (Gomez et al., 2020)	PEAC	Unspecified csDMARDs	52 (E); 58 (V)	Responders (30 (E); 36 (V))	Internal	EULAR response at 6 months	0.92 (O, E); 0.83 (O, V)	0.68 (O, E); 0.59 (O, V)	0.80 (V)
Random forest model consisting of 6 specialised pro-resolving lipid mediators in plasma (Gomez et al., 2020)							0.94 (O, E); 0.87 (O, V)	0.73 (O, E); 0.64 (O, V)	0.79 (V)
Cell profiles									
Cytokine-producing T cells in the blood (Onofrio et al., 2018)	​	Unspecified DMARDs	16 (E)	Non-responders (9 (E))	No validation	EULAR response at 3 months	0.43 (E, E)	0.95 (E, E)	​
Activated T-cell GM-CSF production (cutoff 2.5 pg/mL) (Bystrom et al., 2017)	​	Unspecified TNF inhibitors	97 (E)	Non-responders (76 (E))	No validation	EULAR response at 3 months	0.58 (O, E)	0.73 (O, E)	0.70 [0.56–0.85] (E)
Peripheral type 2–polarized T cell (IL-13+CD4^+^ Tem) (Slauenwhite et al., 2020)	​	MTX	32 (E)	Non-responders (17 (E))	No validation	DAS28-ESR <3.2 at 6 months	​	​	0.74 (E)
CD4:CD8 ratio (Slauenwhite et al., 2020)							​	​	0.75 (E)
Blood pre-DCs (Yamada et al., 2023)	​	ABA	28 (V)	Non-responders (6 (V))	As a validation itself	EULAR response at 6 months	0.81 (O, V)	1.0 (O, V)	0.95

^a^ Combination treatments are denoted by Drug A + Drug B; different treatments received by patients within a cohort are separated by semicolons.

^b^ Study size and the numbers of responders or non-responders are reported for the exploration cohort (E) and, where available, the validation cohort (V).

^c^ Type of validation cohort is classified into internal validation which used the same cohort as the exploration cohort or independent external cohort.

^d^ Specificity and sensitivity were either extracted from the original publication (O) or calculated using raw data from supporting or supplementary files (C). Where no cut-off was specified in the publication but raw data were available, a cut-off was set manually to achieve at least 95% sensitivity (i.e., to detect at least 95% of responders or non-responders), and the estimated specificity (E) was calculated from the raw data.

^e^ Specificity, sensitivity and AUCs are labelled to indicate whether they were derived from the exploration cohort (E) or the validation cohort (V). Where applicable, treatment-specific values are labelled with the corresponding drug abbreviations.

MLM, machine learning model; AUC, area under the receiver operating characteristic curve; ABA, abatacept; ADA, adalimumab; BARI, baricitinib; CZP, certolizumab pegol; ETN, etanercept; GOL, golimumab; HCQ, hydroxychloroquine; IFX, infliximab; LEF, leflunomide; MTX, methotrexate; RTX, rituximab; SSZ, sulfasalazine; TCZ, tocilizumab; TFB, tofacitinib.

Finally, we highlight five key reasons why precision medicine in RA has proved so challenging. (i) RA is highly heterogeneous at the immunological level, which reduces the likelihood that any single biomarker will perform well across most patients (Triaille et al., 2025). (ii) Although technologies have advanced rapidly, they do not yet support the routine use of high-dimensional multi-omics models in a cost-effective and clinically accessible way. (iii) There is still no consensus within rheumatology community on a standardised definition of “response” for predictive biomarker research. (iv) Researchers may be reluctant to actively validate biomarkers and predictive models reported previously, partly because “negative” findings are perceived as less publishable. (v) Finally, the idealised scenario in which patients remain on a fixed therapy for a defined period is often unrealistic, as clinicians are obliged to adjust treatment over time in response to patients’ needs.
